# Complete Regression of an 8-cm Desmoid Fibromatosis After Treatment With Tamoxifen

**DOI:** 10.7759/cureus.37431

**Published:** 2023-04-11

**Authors:** Ryosuke Suzuki, Yusuke Taki, Kazumori Arai, Shinsuke Sato, Masaya Watanabe

**Affiliations:** 1 Department of Gastroenterological Surgery, Shizuoka General Hospital, Shizuoka, JPN; 2 Department of Pathology, Shizuoka General Hospital, Shizuoka, JPN

**Keywords:** gastroenterological surgery, estrogen receptor, selective estrogen receptor modulator, tamoxifen, desmoid fibromatosis

## Abstract

We report a case of a relatively large desmoid fibromatosis that responded completely to tamoxifen as a single drug therapy. A 47-year-old Japanese man underwent laparoscopy-assisted endoscopic submucosal dissection for a duodenal polyp. He developed postoperative generalized peritonitis and underwent an emergency laparotomy. Sixteen months after the surgery, a subcutaneous mass was found on the abdominal wall. Biopsy of the mass revealed estrogen receptor alpha-negative desmoid fibromatosis. The patient underwent total tumor resection. Two years after the initial surgery, he was found to have multiple intra-abdominal masses, with the largest mass measuring 8 cm in diameter. Biopsy revealed fibromatosis, as in the case of the subcutaneous mass. Complete resection was impossible due to the proximity of the duodenum and superior mesenteric artery. Tamoxifen was administered for three years, resulting in complete regression of the masses. No recurrence was observed for the following three years. This case indicates that relatively large desmoid fibromatosis can be successfully treated with a selective estrogen receptor modulator alone and that its effect is not dependent on the estrogen receptor alpha status of the tumor.

## Introduction

Desmoid fibromatosis (DF) is a mesenchymal tumor that exhibits aggressive local growth but lacks metastatic potential. Local growth of DF causes pain, limitations in mobility, impairment of organ function, and bowel obstruction [1]. The degree of symptoms varies from case to case, and clinical factors such as age (≤37 years), tumor localization (extra-abdominal), and tumor size (>7 cm) are reported to be associated with poor clinical outcomes [2]. A wide range of treatment options has been reported for DF, including watchful waiting, medical therapy, cryoablation, radiation, and surgical resection [3]. However, treatment strategies should be based on the malignant potential of DF and the patient’s background. Here, we report a case of unresectable estrogen receptor alpha (ERα)-negative DF measuring 8 cm in diameter. These masses regressed completely after administering tamoxifen, one of the selective estrogen receptor modulators (SERMs), as a single therapy for three years.

## Case presentation

A 47-year-old Japanese man was referred to our hospital for a duodenal polyp detected on esophagogastroduodenoscopy. His medical history included hyperlipidemia and colorectal polyps. The patient had no family history of familial adenomatous polyposis. A biopsy of the polyp revealed an adenoma. Laparoscopy-assisted endoscopic submucosal dissection (ESD) was performed. Histological examination of the resected specimen revealed tubular adenocarcinoma in the duodenum. He developed ESD-related duodenal perforation and generalized peritonitis on the ninth postoperative day. We performed an emergency laparotomy, closed the perforated duodenum, and placed drains. He was treated percutaneously for a residual abscess and discharged 42 days after the initial surgery.

**Figure 1:**
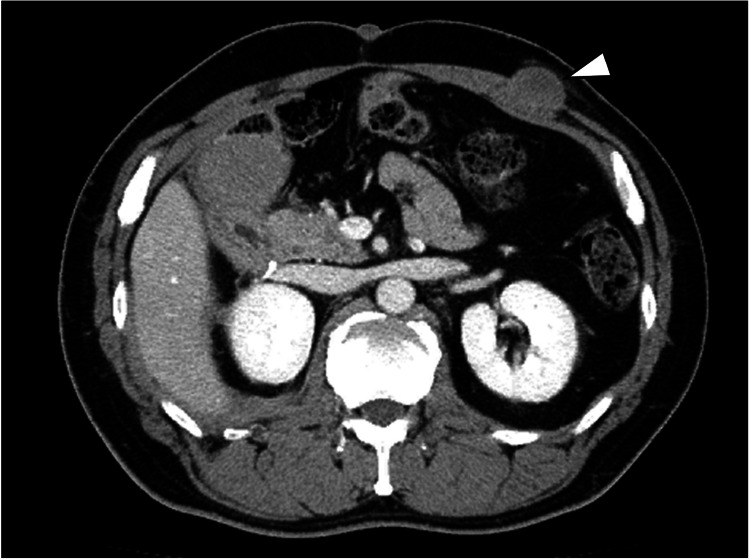
Computed tomography image of the subcutaneous mass. Computed tomography image showing a 25-mm subcutaneous mass (white arrowhead) in the abdominal wall.

The patient underwent outpatient follow-up, including periodic computed tomography (CT) examinations. Sixteen months after the surgery, a subcutaneous mass was noticed on the abdominal wall of the left hypochondrium, which was at the site of the surgical wound (Figure 1).

**Figure 2:**
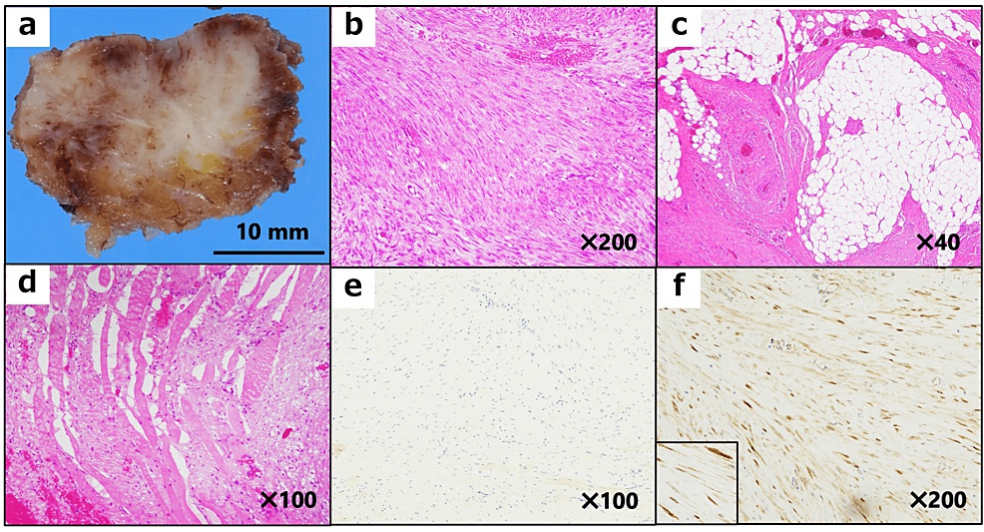
Pathological examination of the initial mass in the abdominal wall. (a) A solid mass with a whitish-cut surface. (b) Long sweeping fascicles consist of uniform spindle-shaped cells without cellular atypia and nuclear hyperchromasia (hematoxylin and eosin stain). (c, d) The mass shows infiltration of adjacent adipose tissue (c) and striated muscular tissue (d). (e, f) The tumor is negative for estrogen receptor alpha (skeletal muscles are non-specifically positive) (e) but positive for β-catenin (f).

A needle biopsy of the mass revealed DF. The patient underwent complete tumor resection. Immunohistochemical analysis indicated that the tumor was negative for ERα and progesterone receptor (PR) but positive for β-catenin (Figure 2).

**Figure 3:**
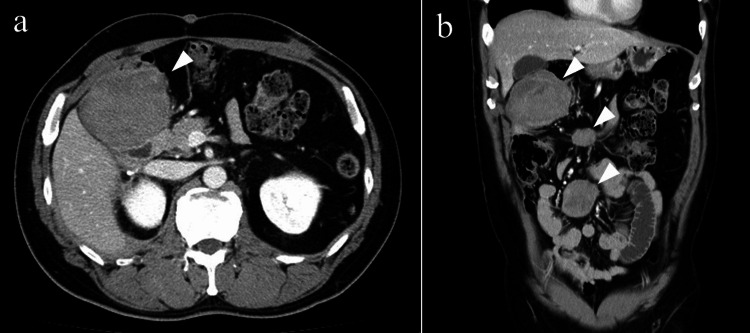
Computed tomography image of the abdominal masses Axial (a) and coronal (b) abdominal computed tomography images showing multiple intra-abdominal masses (white arrowhead). The largest one is below the gallbladder, measuring 8 cm in diameter.

Two years after the initial surgery, multiple intra-abdominal nodules were detected on a CT scan (Figure 3), the largest of which was 8 cm in diameter.

**Figure 4:**
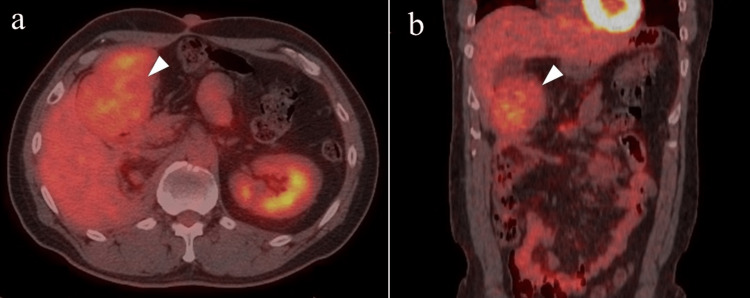
Positron emission tomography image of the abdominal mass. Axial (a) and coronal (b) positron emission tomography images showing minimal uptake (maximum standard uptake value: 4.1) in the mass below the gallbladder (white arrowhead).

The uptake of 18F-fluorodeoxyglucose in these masses during positron emission tomography imaging was low, with a maximum standardized uptake value of 4.1 (Figure 4).

A needle biopsy of the mass revealed fibromatosis, similar to that in the subcutaneous mass. Immunohistochemical analysis of the mass showed negative results for ERα and PR, similar to the findings for the previous mass. Complete resection of these masses was impossible because of the physical proximity to the duodenum and superior mesenteric artery. Therefore, the patient received 20 mg of tamoxifen per day as hormone therapy for DF. After eight months of tamoxifen administration, all masses had reduced size on CT. After 38 months of tamoxifen administration, a CT scan revealed that all tumors had disappeared. Therefore, tamoxifen treatment was terminated after 40 months. No recurrence of the tumors was observed on CT during the three-year follow-up.

## Discussion

We report a case of multiple DF without ERα treated successfully with an SERM alone; three years of follow-up confirmed complete remission (CR). This case report highlights two important clinical issues. First, relatively large abdominal DF can be treated with an SERM alone. Second, the expression of ERα in the tumor may not be related to the effectiveness of SERMs.

SERMs have been used as systemic therapy for DF. In a review of 168 patients with DF, the overall response rate (ORR) to SERMs was 51%. However, this review did not report the CR rate [4]. In a large single-center observational study of 134 patients with DF treated with SERMs, the ORR for SERMs was 85.1%, and the CR rate was 15.9%. The mean period to respond with at least stable disease was 14.9 months [5]. Because the CR rate is relatively low and the treatment period is long, establishing a prediction of treatment efficacy with SERMs is essential. The risk factors for poor prognosis of DF include age (≤37 years), tumor size (>7 cm), and tumor site (extra-abdominal) [2,6]. Although this patient had one risk factor (tumor size of 8 cm), he achieved CR with an SERM alone.

The literature on the association between hormone receptor expression and clinical outcomes is limited. Most studies have shown that DF is ERα negative [7-10]. These results indicate that the effects of SERMs may not be attributable to ERα. Several studies have analyzed ERβ expression in DF. With a 10% cut-off, ERβ has been reported to be expressed in 7.4%-95% of DF [8,9,11], and with a 1% cut-off in 82%-90% [7,12]. However, one of these studies, including 12 cases of DF treated with tamoxifen, suggests that ERβ expression is not associated with response to tamoxifen [7], in agreement with other reports [8,10]. In our case, the DF did not express ERα, but the patient achieved CR with tamoxifen. Immunohistochemical analysis of ERβ was not performed because it was not commercially available. Based on the previous reports, ERβ expression might not be crucial in predicting the response to tamoxifen. Taken together, the analysis of ER expression may not be necessary before the administration of SERMs for patients with DF.

Although the surgery was the primary treatment modality for DF before 2000 [3], non-operative management approaches such as observation, anti-hormonal therapies, tyrosine kinase inhibitors (TKIs), chemotherapy, and radiotherapy have been introduced more recently [13]. One of the largest studies comparing immediate surgery with an initial watchful-waiting approach showed no significant difference in the two-year event-free survival rate (53% vs. 58%, p = 0.915) [14]. Sobczuk et al. reported similar results in a retrospective study [15]. Some DFs can be effectively treated by non-operative interventions [6,16,17]. Moreover, even after negative margin resection, local recurrence remains an issue in up to 35% of patients [1]. Therefore, non-surgical management should be selected as the first-line approach for patients with DF without poor prognostic factors. While the efficacy of TKIs was proven in randomized controlled trials (RCTs) [18,19], that of SERMs has not yet been proven in RCTs. The efficacy of SERMs based on retrospective studies and evidence showing limited side effects highlight this option despite the shortage of RCT studies. In this case, we started with SERMs because the patient had no urgent conditions.

## Conclusions

We encountered a case of ERα-negative DF, measuring 8 cm in diameter, treated with an SERM alone. This case study indicates that DF with one poor prognostic factor (tumor size >7 cm) can be effectively treated with non-surgical interventions and that the absence of ERα in the tumor does not predict the efficacy of SERMs. Even in relatively large DF, SERMs are one of the most effective therapeutic options with few side effects.
